# Prokinetic drugs that stimulate 5-HT_4_-serotonin receptors in the human atrium

**DOI:** 10.1007/s00210-026-05659-1

**Published:** 2026-07-22

**Authors:** Joachim Neumann, Uwe Kirchhefer, Britt Hofmann, Ulrich Gergs

**Affiliations:** 1 Institute for Pharmacology and Toxicology, Medical Faculty, Martin-Luther University Halle-Wittenberg, Magdeburger Straße 4, 06097 Halle (Saale), Germany; 2 Department of Cardiac Surgery, Mid-German Heart Centre, University Hospital Halle, Ernst-Grube Straße 40, 06097 Halle (Saale), Germany; 3 Institute for Pharmacology and Toxicology, Medical Faculty, Münster University, Domagkstraße 12, 48149 Münster, Germany

**Keywords:** Prokinetics, Human atrium, Mouse atrium, 5-HT_4_-serotonin receptors

## Abstract

Several gastrointestinal drugs like bromopride, cinitapride, cisapride, clebopride, felcisetrag, metoclopramide, mosapride, naronapride, prucalopride, renzapride, tegaserod, velusetrag, and zacopride are probably agonists at 5-HT_4_-serotonin receptors in the intestine. Some of these drugs do not act selectively only on 5-HT_4_-serotonin receptors. Instead, most of them also stimulate or inhibit other serotonin receptors. In addition, they sometimes stimulate or inhibit a wide variety of additional targets such as histamine receptors, adrenoceptors, dopamine receptors, monoamine transporters, or potassium channels. We review here to which extent these drugs are potent and/or effective agonists or antagonists at human cardiac 5-HT_4_-serotonin receptors. We address the possibility that at therapeutic concentrations some of these drugs may induce cardiac side effects via 5-HT_4_-serotonin receptors. Plasma concentrations of these drugs can sometimes be elevated by drug/drug interactions such that cardiac side effects become more likely. Moreover, we address the question whether such effects occur at the human cardiac atrium or at the human ventricle. We will focus on inotropic effects, but for completeness also address potential effects on the sinus node. We will present our view where there are remaining pre-clinical or clinical research needs. One may repurpose some of these drugs to act on cardiac 5-HT_4_-serotonin receptors and discuss for which indications this may offer additional benefits.

## Introduction: serotonin formation and function in the gut

**Fig. 1:**
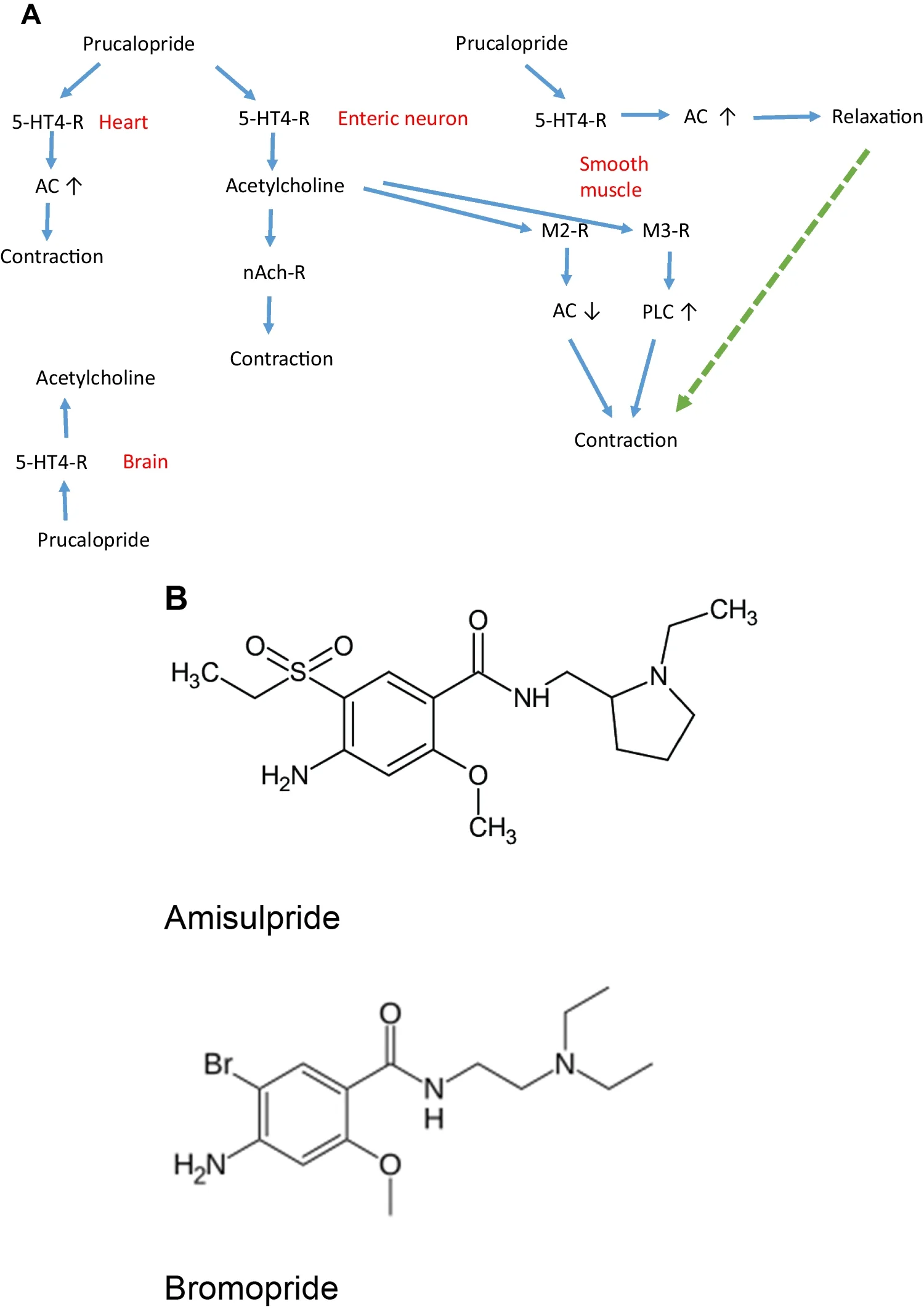

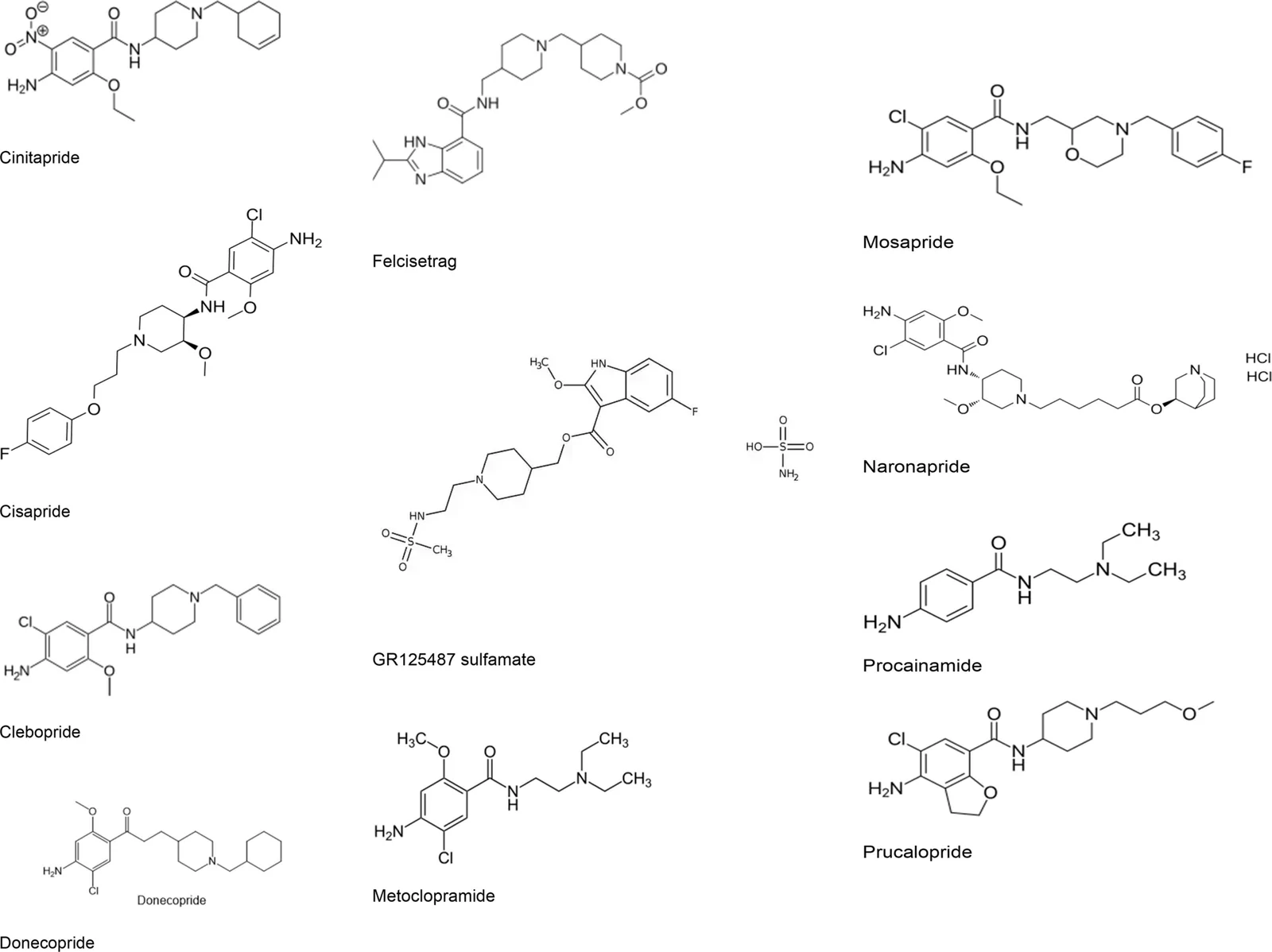

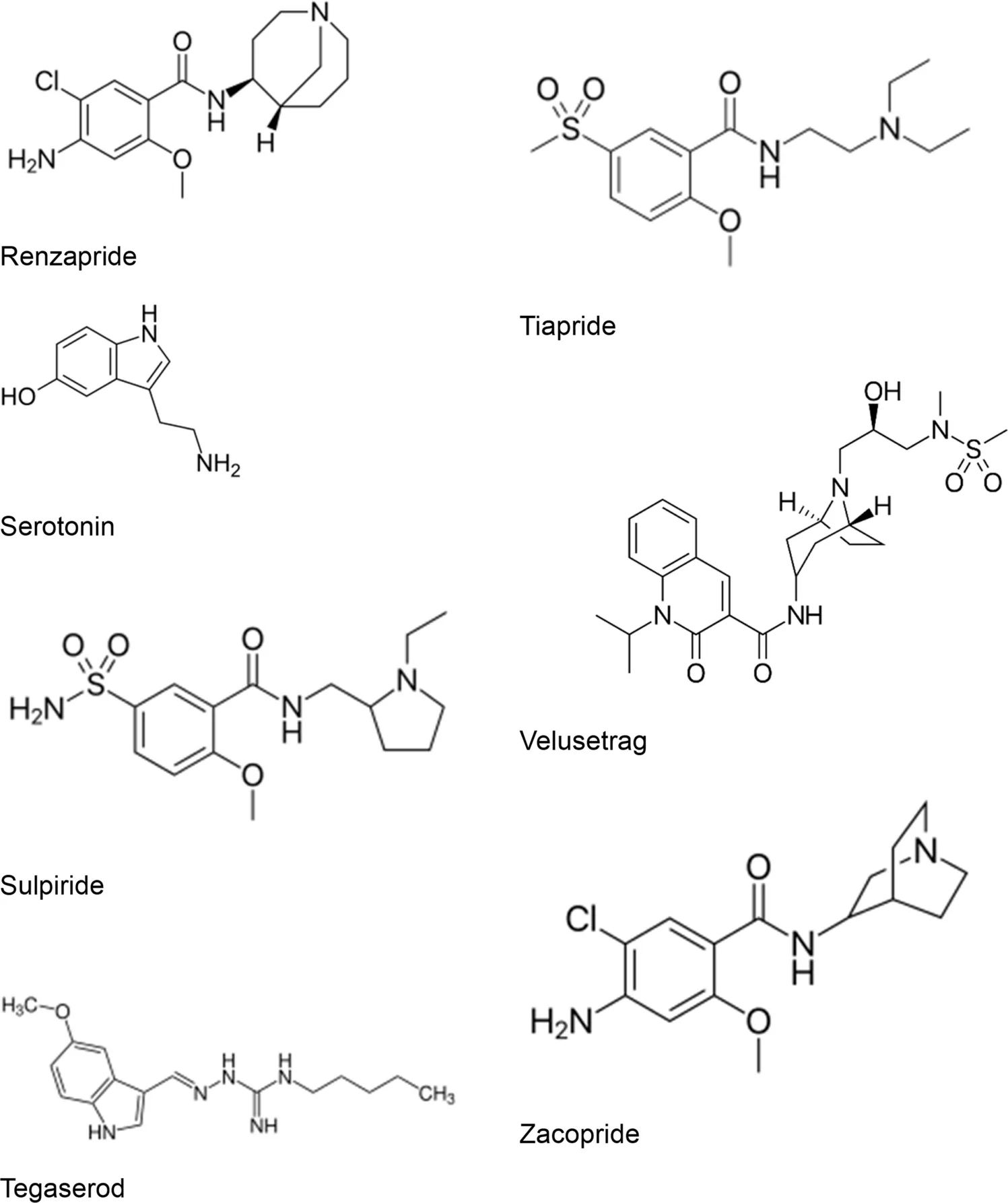
**A** Tissue specific effects of 5-HT_4_-serotonin receptors. As an example of a rather specific 5-HT_4_-serotonin receptor (5-HT_4_-R) agonist, prucalopride, is seen with its action in the heart, in the gut on enteric cells and smooth muscle cells and in the brain. In the heart, 5-HT_4_-R lead to stimulation of adenylyl cyclase (AC). This finally leads to a contraction of the cardiac tissue, a positive inotropic effect but also to a positive inotropic effect and potentially to cardiac arrhythmias. In the brain under the same conditions serotoninergic nerves lead to a release of stored acetylcholine. A similar effect happens in the gut: released acetylcholine from enteric motor neurons following stimulation of 5-HT_4_-serotonin receptors induces smooth muscle contraction via muscarinic acetylcholine receptors. In addition, stimulation of 5-HT_4_-serotonin receptors may facilitate the release of acetylcholine from enteric interneurons, which in turn excites cholinergic motor neurons via nicotinic acetylcholine receptors (nAch-R), ultimately resulting in smooth muscle contraction mediated by muscarinic acetylcholine receptors (Rueda Ruzafa et al. 2021). However, 5-HT_4_-R on smooth muscle cells of the bowel activate AC. This leads finally to relaxation of the smooth muscle in the adjacent bowel. However, acetylcholine via M_2_-musarinic receptors (M2-R) can presumably inhibit the activity of AC. This would counteract the effect due to an activation of AC via direct stimulation of AC by 5-HT_4_-R. In addition, acetylcholine can stimulate in smooth muscle cells the activity of phospholipase C (PLC) via M_3_-muscarinic receptors (M3-R). PLC leads to contraction of the smooth muscle cells in the gut. In a species- and region-specific manner, 5-HT_4_-R can either simply lead to relaxation of segments of the intestine or relax segments of the intestine that have been contracted by acetylcholine or can solely lead to contraction of the segments of the intestine. The green arrow indicates that 5-HT4R-mediated relaxation of the gut muscle layers might counteract a contraction of the gut muscle layers. **B** Structural formulae of the drugs of interest. The drugs are presented in alphabetical order. One may point out that bromopride and metoclopramide only differ in the halogen atom. Most drugs show a clear benzamide function. The lead compound for the benzamide is procainamide, an antiarrhythmic drug. An indole ring is prominent in serotonin, felcisetrag and tegaserod. Velusetrag presents a unique structure: no benzamide function and no indole ring are found in velusetrag. A sulfonic function is a common feature in amisulpride, sulpiride and tiapride which are used as antipsychotic drugs. Halogen atoms are found in bromopride, cisapride, clebopride, metoclopramide, mosapride, naronapride, prucalopride, and renzapride

About 80–95% of the total serotonin (5-hydroxytryptamine, 5-HT) in the human body is formed and stored in the gut (Gershon and Tack 2007). In addition, about 5% of serotonin is formed in the brain (Gershon and Tack 2007). Serotonin does not pass or at best poorly passes through the blood brain barrier (Gershon and Tack 2007). In the gut, serotonin is mainly found in specialized cells in the intestinal mucosa called by early histologists as enterochromaffin cells (Gershon and Tack 2007). Indeed, the gut and not the blood was the first location where serotonin was detected (called enteramine by Erspamer 1937). Enterochromaffin cells do not only contain serotonin but a host of peptides such as vasoactive intestinal peptide (VIP) or pituitary adenylate cyclase-activating peptide (PACAP), which may induce relaxation and thus can act as functional antagonists to constrictive effects of serotonin on bowel segments (Seretis et al. 2004, Germano et al. 2009). Only a small amount of serotonin enters the body through the food we eat. Some serotonin is formed in the lumen of the bowel by bacteria and has gained a lot of research efforts in recent years (e.g., O’Mahony et al. 2015). The bulk of serotonin is formed in the gut in the afore-mentioned enterochromaffin cells (Gershon and Tack 2007). This synthesis of serotonin in the enterochromaffin cells in the gut (but also in cardiomyocytes) starts from the essential amino acid tryptophan via tryptophan hydroxylase 1. In contrast, in nerve cells in the brain but also in nerve cells in the gut, tryptophan hydroxylase 2 is used for formation of serotonin (Kendig and Grider 2015). Tryptophan hydroxylases are the rate limiting enzymes for the synthesis of serotonin in, e.g., enterochromaffin cells or nerve cells (Beattie and Smith 2008). Thereafter, the product, 5-hydroxy-tryptophan, is decarboxylated by another, abundant enzyme, an unspecific decarboxylase, and thus finally serotonin is formed in cells (Neumann et al. 2023a). In response to, e.g., pressure by propulsion of food in the intestine, serotonin is released from these intestinal enterochromaffin cells, probably mediated via pressure-sensitive receptors in the mucosa (Bülbring and Lin 1958; Bülbring and Crema 1959; Gershon 2004). Serotonin released from enterochromaffin cells acts probably as a local paracrine messenger: serotonin diffuses to nearby nerve endings in the gut (Fig. 1A). At physiological pH, a nitrogen atom in serotonin is protonated and therefore serotonin cannot easily pass biomembranes via diffusion. This passage occurs via specialized transporter proteins (SERT) into the cytosol of, e.g., thrombocytes of the blood that pass through the wall of the bowel (Mawe and Hoffman 2013). This serotonin in thrombocytes is the main source of the serotonin which we find, e.g., in the human heart (Mawe and Hoffman 2013). Taking these mechanisms into account, one notes in patients, with irritable bowel disease and constipation, in the blood low concentrations of serotonin (Mawe and Hoffman 2013). This could mean that insufficient serotonin is formed in the enterochromaffin cells in the bowel to activate intestinal serotonin receptors in this specialized group of patients. This may be a logical basis to give drugs that stimulate intestinal 5-HT_4_-serotonin receptors in diseases with impaired bowel motility (Mawe and Hoffman 2013). The local concentrations of serotonin in the gut may be quite high (milli molar concentrations) upon the release of serotonin from enterochromaffin cells (Gershon and Tack 2007). Hence, one has argued that in order to protect intestinal 5-HT_4_-serotonin receptors against prolonged and excessive stimulation by local serotonin in the bowel, local inactivation of serotonin by degradation (via monoamine oxidases and transferases) occurs. Moreover, the serotonin levels near intestine membranes are reduced by SERT-driven uptake into the enterochromaffin cells or in local enterocytes (Gershon and Tack 2007).

## Serotonin receptors

Serotonin can increase the motility of the isolated, beating gut (e.g., guinea pig ileum: Gaddum and Picarelli 1957). This increase in motility can be divided into at least two steps with different receptors involved. In guinea-pig ileum, serotonin increases contraction via the so-called M-receptors in the nervous tissue and via so-called D-receptors in the intestinal muscle (Gaddum and Picarelli 1957). About 30 years later, it became clear that these contractile effects of serotonin in the gut via the M-receptors are indeed mediated by 5-HT_4_-serotonin receptors (Dumuis et al. 1989). The D-receptors in the guinea-pig ileum were suggested to be identical or similar to the 5-HT_3_-serotonin receptors (Dumuis et al. 1989). The 5-HT_4_-serotonin receptor was first cloned in the year 1995 (rat: Gerald et al. 1995, human: Blondel et al. 1997; Claeysen et al. 1997). This convinced readers that the 5-HT_4_-serotonin receptor forms a distinct receptor class (Bockaert et al. 2004). Over the years, it became clear that the 5-HT_4_-serotonin receptor is coded by a complicated gene in humans with at least 38 exons, leading to at least eight splice variants (Bender et al. 2000, Bockaert et al. 2004). The first cloning paper already described two splice variants now called 5-HT_4a_-serotonin receptors and 5-HT_4b_-serotonin receptors which are the main splice variants in the human heart (Gerald et al. 1995, Bockaert et al. 2004). 5-HT_4_-serotonin receptors are presynaptic on cholinergic nerve terminals in the gut (Fig. 1A, Liu et al. 2005). Using mice where the 5-HT_4_-serotonin receptor is removed and is substituted by a fluorescent protein (knock-in), one detected the 5-HT_4_-serotonin receptors in nearly all cells in the mucosa of the colon including enterochromaffin cells (Hoffman et al. 2012). More than two splice variants of the 5-HT_4_-serotonin receptor were reported not only in the human gut but also the human heart (Läer et al. 1998, Bach et al. 2001, De Maeyer et al. 2008, Kaumann and Levy 2006, Barnes et al. 2021). Currently, we distinguish seven serotonin receptor families (Barnes et al. 2021). Each family of serotonin receptors encompasses several splice variants. With the exception of the 5-HT_3_-serotonin receptor (which acts as an ion channel), all serotonin receptors couple via guanosine triphosphate-binding proteins (G-proteins) to one or more effector proteins. 5-HT_4_ serotonin receptors were the first serotonin receptors that were shown to stimulate the activity of adenylate cyclase via stimulatory G-proteins. As a consequence, 5-HT_4_-serotonin receptors increased cellular levels of 3′:5′ cyclic adenosine monophosphate (cAMP) (Dumuis et al. 1989, Fig. 1A, Barnes et al. 2021). Currently, two more serotonin receptors are noted to stimulate cAMP formation, namely, 5-HT_6_- and 5-HT_7_ serotonin receptors (Barnes et al. 2021), which will not be discussed here, but which are the subject of active research in the gut and in the heart. 5-HT_4_-serotonin receptors are expressed in many organs. In the present context, we only consider their expression in the gut, the brain, and the heart (Fig. 1A). For more details on other tissues, we refer the interested reader to excellent recent reviews (e.g., Barnes et al. 2021). There are some parts of the human intestine and regions of the heart that express only specific splice variants of the 5-HT_4_-serotonin receptor. However, we do not yet fully understand the purpose of these splice variances. Any differences in the potencies of agonists and antagonists at splice variants of the 5-HT_4_-serotonin receptors are minute, both in the gut and in the heart of man, with the possible exception of renzapride and prucalopride (Kaumann and Levy 2006). 5-HT_4_-serotonin receptors are expressed in both enteric neurons and in smooth muscle cells of the gut (Galligan 1996; Tack et al. 2012; Baxter et al. 1991). When serotonin activates neuronal 5-HT_4_-serotonin receptors in the gut, then a prokinetic activity ensues in the gastrointestinal tract (Gershon 2005). This contraction comes probably about in the following way (Fig. 1A): serotonin can enhance the release and amplify the action of acetylcholine in the mammalian gut (Tonini et al. 1989, Galligan 1996, Gershon and Tack 2007, De Maeyer et al. 2008, Tack et al. 2012, Fig. 1A). Acetylcholine leads to contraction of the circular smooth muscle in the gut via M_2_-muscarinic receptors and/or M_3_-muscarinic receptors (Olsson and Holmgren 2001, Wells and Blennerhassett 2005, Fig. 1A). In addition, contraction of longitudinal smooth muscle likewise occurs in the gut. This interaction of longitudinal contractions and circular contractions in the intestine is thought to underlie the transit of food in an oral to aboral fashion (Beattie and Smith 2008). On the one hand, serotonin releases stored acetylcholine from cells in the gut. This released acetylcholine can then alter the motility in various parts of the gastrointestinal tract usually in the form of contractions (De Maeyer et al. 2008, Tack et al. 2012; Zhang et al. 2020). On the other hand, serotonin via 5-HT_4_-serotonin receptors can increase cAMP which can lead to relaxation of smooth muscle in the gut (Fig. 1A). This relaxation is augmented by other receptor agonists that increase cAMP in smooth muscle cells such as PACAP and VIP (Olsson and Holmgren 2001).

## Gastrointestinal drugs and serotonin receptors

Usually, potent 5-HT_4_-serotonin receptor agonists are prokinetic drugs in mammals including humans (Barnes et al. 2021; Tack et al. 2012). More specifically, stimulation of 5-HT_4_-serotonin receptors relaxed isolated spontaneously contracting intertaenial circular muscle strips from the human colon (Tam et al. 1994). Serotonin stimulated cAMP-levels in human colonic circular smooth muscles via 5-HT_4_-serotonin receptors (McLean and Coupar 1996). This suggests that 5-HT_4_-serotonin receptors in the human gut use, at least in part, cAMP as their second messenger as they do in other organs like the heart or the brain (Kaumann et al. 1991, McLean and Coupar 1996). A role for 5-HT_4_-serotonin receptors in the gut was also supported by another, genetic, study: the gastric emptying, the small intestinal transit, and the large intestinal transit were reduced in mice with constitutive deletion of the 5-HT_4_-serotonin receptor compared to wild-type littermate mice (Liu et al. 2009). This shows that 5-HT_4_-serotonin receptors are not only expressed but have a stimulatory function on motility in the gut, at least in mice and thus hypothetically, also in humans. The role of 5-HT_4_-serotonin receptors is not confined to regulation of mechanical functions in the gut. Moreover, stimulation of 5-HT_4_-serotonin receptors also promotes chloride secretion in human intestinal cells (Borman and Burleigh 1997). A certain gut disease namely irritable bowel syndrome is painful. In this context, it may be noteworthy that stimulation of 5-HT_4_-serotonin receptors in the gut can diminish visceral hyperalgesia in patients suffering from the irritable bowel syndrome (Greenwood-Van Meerveld et al. 2006).

### Chemical structures of the drugs of interest

The drugs of interest in this review belong chemically to several larger or smaller groups (cf. Langlois and Fischmeister 2003, Castriconi et al. 2018, Barnes et al. 2021). Based on their main functional groups (some selected drugs are in Fig. 1B), one might separate them into benzimidazolones, benzamides (bromopride, cinitapride, cisapride, clebopride, metoclopramide, naronapride, prucalopride, renzapride), benzoic esters, aryl ketones, indole carboxylates, carboxamides (velusetrag), and serotonin derivatives (tegaserod) (Langlois and Fischmeister 2003). The development of these drugs was initially serendipitous, e.g., for metoclopramide or cisapride. In general, older benzamides show usually moderate affinity and poor selectivity for the 5-HT_4_-serotonin receptors (Tack et al. 2012, Table 4). Only later a rational design based on binding affinity to 5-HT_4_-serotonin receptors has gained some success and led to velusetrag, naronapride and prucalopride as prominent examples (cf. Table 1 and Table 2).

**Table 1:** Cardiac inotropic and chronotropic effects of the drugs of interest

Drug	RA 5-HT_4_-TG	Porcine RA	LA-5-HT_4_ -TG	Porcine LA	HAP	HAP + PDE	Human ventricle	Human ventricle + PDE	Reference
Bromopride	+ ^1^	ND	+ ^1^	ND	-^1^	+ ^1^	ND	ND	1:Neumann et al. 2025a
Cinitapride	+ ^1^	ND	+ ^1^	ND	-^1^	+ ^1^	ND	ND	1:Neumann et al. 2024c
Cisapride	+ ^5^	+ ^2,4^	+ ^5^	+ ^3^	+ ^1,3,6^	ND	ND	ND	1:Kaumann et al. 1991 2:Kaumann 1990 3:Conlon et al. 2018 4:Kaumann et al.^1990^ 5:Keller et al. 2018 6:Chai et al. 2012
Clebopride	+ ^1^	ND	+ ^1^	ND	-^1^	+ ^1^	ND	ND	1:Rayo Abella et al. 2025b
Felcisetrag	+ ^1^	ND	+ ^1^	ND	-^1^	+ ^1^	ND	ND	1:Rayo Abella et al. 2025a
Metoclopramide	+ ^1^	+ ^2^	+ ^1^	+ ^2^	+ ^1^	+ ^1^	ND	ND	1:Neumann et al. 2021 2:Medhurst and Kaumann 1993
Mosapride	+ ^1^	ND	+ 1	ND	-^1^	+ ^1^	ND	ND	1:Neumann et al. 2024b
Naronapride	+ ^1,2^	ND	+ ^1,2^	ND	-^2^	+ ^1,2^	+ ^1^	ND	1:Schmidt et al. 2025 2: Neumann et al. 2024a, b, c, d
Prucalopride	+ ^1^	+ ^2,3^	+ ^1^	+ ^2,3^	+ ^1,3^	ND	ND	ND	1:Keller et al. 2018 2:De Maeyer et al. 2006 3:Conlon et al. 2018
Renzapride	ND	+ ^1,2^	ND	ND	+ ^3^	ND	ND	ND	1:Kaumann 1990 2:Medhurst and Kaumann 1993 3:Kaumann et al. 1991
Serotonin	+ ^1^	+ ^1^	+ ^1^	+ ^1^	+ ^1,5^	+ ^1^	-^1^ + ^2,3,4^	+ ^2,3^	1:Kaumann and Levy 2006 2:Brattelid et al. 2004, 3:Brattelid et al. 2007 4:Qvigstad et al. 2005 5:Chai et al. 2012
Tegaserod	+ ^1^	+ ^2^	+ ^1^	+ ^2^	+ ^1,3^	+ ^1^	ND	ND	1:Hesse et al. 2024 2:De Maeyer et al. 2006 3:Chai et al. 2012
Tiapride	-	ND	+	ND	-	-	ND	ND	Unpublished
Velusetrag	+ ^1^	ND	+ ^1,3^	ND	+ ^1,2^	+ ^1,2^	ND	ND	1:Pauls et al. 2026 2:Neumann et al. 2024c 3:Schmidt et al. 2025
Zacopride	+ ^1^	+ ^2,3^	+ ^1^		+ ^1^	+ ^1^	NIE^4^	ND	1:Neumann et al. 2024a 2:Villalón et al. 1991 3:Eglen et al. 1993 4:Elnakish et al. 2017

Drugs appear in alphabetical order. (+): positive inotropic effect only in failing human ventricle strips. (-): no effect. RA 5-HT_4_-TG: increase in frequency in isolated beating right atrial preparations from mice with cardio-selective overexpression of the human 5-HT_4_-serotonin receptor. LA 5-HT_4_-TG: increase in force of contraction in isolated electrically stimulated left atrial preparations from mice with cardio-selective overexpression of the human 5-HT_4_-serotonin receptor. Porcine RA: increase in frequency in isolated spontaneously beating right atrial preparations from pigs or piglets. Porcine LA: increase in force of contraction in isolated electrically stimulated left atrial preparations from pigs or piglets. Human: + : increase in force of contraction in the absence or presence (+ PDE) of phosphodiesterase inhibitors in isolated electrically stimulated right atrial preparation from human atrium. HAP: force of contraction in the absence or presence (+ PDE) of phosphodiesterase inhibitors in isolated electrically stimulated right atrial preparation from human atrium. Human ventricle + : increase in force of contraction in the absence or presence (+ PDE) of phosphodiesterase inhibitors in isolated electrically stimulated ventricular preparations from the human heart. *ND*, not done. *: approved drug, at least in one country worldwide. The other drugs are discontinued or waiting for approval or of unknown status

**Table 2:** Pharmacokinetic parameters and additional receptor interactions of the drugs of interest

Drug	Half-life	Peak plasma concentration	Metabolism	Receptor binding	Reference
Bromopride*	p.o 4h^3^	p.o.: 0.1–1 µM^1^	CYP^1^	D_2_-dopamine re-ceptor antagonist^2^	1:Dunne et al. 2013 2:Meltzer et al. 1983 3:Brodie et al. 1986
Cinitapride*	8h^1^	2.2 nM^1,3^	CYP^2^	D_2_-antagonist^1^ 5-HT_2A_-antagonist^1^ 5-HT_3_-antagonist^1^	1:Robert et al. 2007 2:Marquez et al 2011 3:Campodonico et al. 2022
Cisapride*	7–10 h^1,5^	92 nM^5^	CYP3A4^2^	NET^4^ 5-HT_2C_^4^ 5-HT_1D_^4^ 5-HT_1A_^4^ SERT4^4^ 5-HT_2A_^2,4^ Αlpha1-R^2^ 5-HT_3_^2,4^ 5-HT_2B_^2^ D_2_-receptor^2,4^ hERG^2,3,4^	1:McCallum et al. 1988 2:Sanger 2009 3:Mendzelevski et al. 2011 4:Conlon et al. 2018 5:Kivistö et al. 1999
Clebopride*	3–6 h^6^	6.7–13 nM^6^	CYP3A4^5^	5-HT_3_-R antagonist^2^ 5-HT_4_-R^1^ hERG inhibition^4^ Binding to D_1_-, D_3_-, D_4_^3^ D_2_-dopamine re-ceptor antagonist^1^	1:Tonini et al. 2004 2:Takeda et al. 1991 3:Einsiedel et al. 2003 4:Kim et al. 2007 5:Sun and Scott 2011 6:Robinson et al. 1991
Felcisetrag	70 h^1^	7 nM^2^	CYP3A4^2,3^		1:Pulsalkar et al. 2022 2:Czerniak et al. 2022 3:Chen et al. 2022
Metoclopramide*	3–5 h^1^	423 nM^1^	CYP2D6^2^	D_2_-dopamine antagonist	1:Bateman 1983 2:Livezey et al. 2014
Mosapride*	2.4 h^5^	0.67 µM^3^	CYP3A4^4^	5-HT_3_ antagonist^1^ ↑hERG^2^	1:Park and Sung 2019 2:Carlsson et al. 1997 3:Endo et al. 2002 4:Katoh et al. 2003 5:Kim et al. 2012
Naronapride	6h^1^	24 nM^1^	Little metabolism^1^		1:Bowersox et al. 2011
Prucalopride*	24–30 h^1^	12 nM^1^	Little metabolism^1^	α_2C_-adrenoceptor^2^ D_2_-dopamine antagonist^2^ 5-HT_3A_^2^ 5-HT_2C_^2^	1:Frampton 2009 2:Conlon et al. 2018
Renzapride	26–36 h^3^	19 nM^1^	CYP3A4^2^	5-HT_3_- antag^2^ 5-HT_2A_- antag^2^ 5-HT_2B_- Antag^2^ 5-HT_2C_- antag^2^	1:Staniforth and Pennick 1990 2:Meyers and Hickling 2008 3:Tack et al. 2012
Tegaserod*	6–10 h^2^	20 nM^2^ 120 nM^3^	CYP2D6^2^ CYP3A^4^	5-HT_1A_-agonist^5,6^ 5-HT_1B_-agonist^5,6^ 5-HT_1D_-agonist^6^ 5-HT_2A_-antag^5^ 5-HT_2B_-antag^5^ 5-HT_1F_^5^ 5-HT_2B_^1,5^ 5-HT_2C_^1,5^ 5-HT_3A_^5,1^ 5-HT_5A_^5^ 5-HT_5_^1^ 5-HT_6_^1^ 5-HT_7_^1,5^ SERT^5^ NET^5^ hERG^1,5^ DAT^5^	1:Beattie et al. 2013 2:Appel-Dingemanse 2002 3:Appel et al. 1997 4:Vickers et al. 2001 5:Conlon et al. 2018 6:De Maeyer et al. 2008
Tiapride*	1.4–5h^1,2^	3.9–7.9 µM^1^	CYP^2,3^	D_3_-antagonist^4^ D_2_-antagonist^2^ D_1_-antagonist^2^	1:Roos et al. 1986 2:Steele et al. 1993 3:Nobilis et al. 2011 4:Scatton et al. 2001
Velusetrag	10–14 h^5^	120 nM^5^	CYP3A4^5^	H_1_R antagonism^1^ hERG^4^	1:Beattie et al. 2013 2:Pauls et al. 2026 3:Smith et al. 2008 4:Long et al. 2012 5:Manini et al. 2010
Zacopride	ND	ND	ND	5-HT_3_ antagonist^1^ K-channel agonist^2^	1:Wu and Cao 2014 2:Liu et al. 2012

The first column indicates the drugs of interest. The second column indicates their plasma half-life. The third column indicates typical plasma molar concentrations in order to facilitate comparison with half maximal effective concentrations (EC_50_-values) in Table 4. Fourth columns indicates possible mechanisms of drug/drug interaction. Therefore isoforms of cytochrome (CYP, where known) are indicated. For instance, drugs without metabolism are increased with deterioration of kidney function, those subject to CYP3A4 metabolism are increased by interacting drugs like erythromycin and those subject to CYP2D6 are increased in the case of the poor metabolizer status. Moreover. for bromopride it seems to be unknown which isoform of CYP is used. The fifth column is meant to indicate data in ligand binding studies. These receptors are relevant on top of the effect on the 5-HT_4_-serotonin receptor. NET: noradrenaline transporter, SERT: serotonin transporter, hERG: human ether-a-gogo-potassium channel or voltage-gated inwardly rectifying potassium channel

### Indications of the drugs of interest

Possible indications for 5-HT_4_-serotonin receptor agonists of interest (Fig. 1B) in gastroenterology encompass treatment of constipation, gastro-esophageal reflux disease, and some forms of the irritable bowel disease (Beattie and Smith 2008), De Maeyer et al. 2008, Waikar et al. 1993). Of note, some drugs that bind to or act on 5-HT_4_-serotonin receptors are used or are intended to be used for indications other than the mobility of the gut. As already mentioned, the 5-HT_4_-serotonin receptors were initially identified and characterized in the brain (Dumuis et al. 1989). Hence, 5-HT_4_-serotonin receptors should serve a purpose in the brain. Thus, it is non implausible that 5-HT_4_-serotonin receptor agonists may be useful to treat neurological or psychiatric diseases. Indeed, 5-HT_4_-serotonin agonists have been suggested as antidepressant drugs (Bockaert et al. 2004). 5-HT_4_-serotonin agonists may lead to weight loss through action on the brain: satiety may be enhanced and craving for food might be reduced (Bockaert et al. 2004, Jean et al. 2007). 5-HT_4_-serotonin receptor agonists have been tested in animal model of Morbus Alzheimer and have had some success in clinical studies of this disease (Liu et al. 2020). A plausible explanation for their use in Morbus Alzheimer can be summarized as follows (similar to the mechanisms in the gut): stimulation of brain 5-HT_4_-serotonin receptors leads to the release of acetylcholine in the brain like in the gut. Increased brain acetylcholine levels might be beneficial: drugs that inhibit the degradation of acetylcholine (cholinesterase inhibitors) show some beneficial effects in some patients suffering from Morbus Alzheimer. Thence, 5-HT_4_-serotonin receptors agonist might become suitable drugs to treat Morbus Alzheimer. Usually, older 5-HT_4_-serotonin receptor agonists have ancillary effects. For instance, one has tested 5-HT_4_-serotonin receptor agonists that with one part of their molecule stimulate 5-HT_4_ serotonin receptors but with another part of their molecule inhibit the activity of cholinesterases. This concept underlies the use of such drugs to treat Morbus Alzheimer (e.g., donecopride: Lecoutey et al. 2014, or MR33317: Neumann et al 2024c). Some drugs that are agonists at 5-HT_4_-serotonin receptors and antagonists at 5-HT_3_ serotonin receptors had some initial success to treat memory deficits (SL 65,0155, caposerod: Bockaert et al. 2004). Most of the older 5-HT_4_-serotonin agonists are also 5-HT_3_-serotonin receptor antagonists (Table 3 and in a concise way in Table 1). That led to their use as antiemetic drugs: stimulation of brain 5-HT_3_-serotonin receptors can induce nausea and vomiting. This mechanism can explain the antiemetic effects of selective 5-HT_3_ serotonin receptor antagonists like ondansetron (Baber et al. 1992). Many 5-HT_4_-serotonin receptor agonists that are used in gastroenterology are also D_2_-dopamine receptor antagonists. One can also make a point that amisulpride, used due its inhibitory action on D_2_-dopamine receptors as an antipsychotic drug, is also a 5-HT_4_-serotonin agonist, in the human heart (Neumann et al. 2026a). Currently, D_2_-dopamine receptor antagonism is thought to be beneficial for the treatment of gastrointestinal mobility disorders in two ways: on the one hand, dopamine inhibits the release of acetylcholine in the gastrointestinal tract (Tonini et al. 2004). Blockage of D_2_-dopamine receptors in the gut enhances the release of acetylcholine in the gut. This acetylcholine would increase the motility of the gut, as mentioned above. This can be beneficial in obstipation. On the other hand, blockage of D_2_-dopamine receptors in the brain contributes to antiemetic effects (Tonini et al. 2004). Clearly, D_2_-dopamine receptor antagonism in the central nervous system is potentially detrimental: it can induce extrapyramidal side effects which are similar to those seen in patients that suffer from Morbus Parkinson. Thence, one has tried over time to develop by chemical modifications of lead compounds new drugs that are selective for 5-HT_4_-serotonin receptor agonism. However, even here while some drugs are very selective at 5-HT_4_-serotonin receptor, they act at low drug concentrations as 5-HT_4_-serotonin agonists and at higher concentration 5-HT_4_-serotonin antagonists (e.g., felcisetrag, Table 1 compared to Table 4). One has suggested that it is dependent on the density of 5-HT_4_-serotonin receptors and the efficiency of the coupling of the receptor to its effector(s) whether a 5-HT_4_-serotonin receptor agonist acts as an agonist or as an antagonist (Bach et al. 2001, Tack et al. 2012). Moreover, tissues devoid of 5-HT_4_-serotonin receptor reserve would not be stimulated by agonists with a low agonist efficacy or by low doses of a high affinity agonist (Tack et al. 2012). This explains conceivably why one detects with some agonists only in the presence of phosphodiesterase inhibitors a positive inotropic effect in the human heart (cf. Table 4). Hence, it is species dependent and also tissue dependent whether a given agonist at the 5-HT_4_-serotonin receptor is a full or partial agonist. For instance, in the guinea pig ileum, cisapride is a partial agonist whereas in the rat esophagus, cisapride is nearly a full agonist (Tack et al. 2012). Also other indications like those in neurology occur (e.g., for amisulpride, sulpiride, tiapride, donecopride, Fig. 1B). If these drugs are mainly used in gastroenterology, one might assume it may be a research need to develop derivatives of these drugs that they are not taken up into the human body. However, in later years, it turned out that these drugs like prucalopride were resorbed (Flach et al. 2016, Frampton 2009). Indeed, it is conceivable that the resorption of these drugs is indispensable for their intestinal action on muscle layers in the gut. Some of the gut action (e.g., antiemesis) of these drugs preferentially occurs in the brain and hence they are sometimes given parenterally to achieve high plasma concentration in order to treat better emesis. They are usually degraded in the liver via cytochromes and then disposed of in the urine or the gut (Table 3). However, there are exceptions to this rule, as some are degraded by other pathways or leave the human body intact (Table 3), as will be discussed to some extent below for individual compounds.

**Table 3:** Pharmacodynamic effects of the drugs of interest

Drug	5-HT_1_	5-HT_2_	5HT_3_	5HT_4_	hERG	D_2_	Trans-porter	Varia
Bromopride				+ -		-		
Cinitapride		-	-	+ -		-		
Cisapride	+	+	+	+	-	-	NET-SERT -	α -
Clebopride			-	+ -	-	-		D_1_,D_3_,D_4_ -
Felcisetrag	No	No	No	+ -	No	No	No	
Metoclopramide			-	+ -	-	-		
Mosapride			-	+ -	-			
Naronapride	No	No	No	+	No	No	No	
Prucalopride	No	-	-	+ -	No	-	No	α_2C_-
Renzapride			-	+				
Tegaserod	+	+	+	+ -	-		DAT, SERT, NET	5-HT_5_ 5-HT_6_ 5-HT_7_
Tiapride				No		-		D_3_
Velusetrag	No	No	No	+ -	-	No	No	H_1_-antagonist
Zacopride			-	+ -				K-channel +

Targets for drug action modified from Table 2 where one can find the corresponding references. The first column indicates the drugs of interest. In the subsequent four columns serotonin receptors are listed. D_2_: the D_2_-dopamine receptor. Thereafter transporters are listed and under varia one finds additional receptors with which an interaction was noted. No: means that one has measured that no stimulation or inhibition of this receptor occurs. A blank space indicates that no published data are available. + : stimulation of a receptor, -: inhibition of a receptor, + -: the drug has agonistic and antagonistic effects at this receptor. Interaction in this table usually means interaction in contractile studies in the isolated gut segments or isolated cardiac preparations. Other abbreviations as in Table 2

### Serotonin receptors in the human cardiac atrium

In our initial work in this area, we first turned to the rat heart and the mouse heart as models for the human heart. However, in the mouse heart, no inotropic or chronotropic effects to serotonin were apparent (Läer et al. 1998, Gergs et al. 2010, Gergs et al. 2013). In the rat heart, we noted a positive inotropic effect of serotonin but also no positive chronotropic effect to serotonin. The positive inotropic effect of serotonin in the rat cardiac preparations was 5-HT_2A_-serotonin receptor-mediated (Läer et al. 1998). Others later observed that in hypertrophied or in severely failing adult rat hearts, 5-HT_4_-serotonin receptors become overexpressed (as mRNA). Serotonin raised force of contraction in cardiac preparations from these hypertrophied rat hearts (Brattelid et al. 2004). However, Kaumann et al. 1990 had convincingly demonstrated that the positive inotropic and also the proarrhythmic effects of serotonin in the isolated human atrium and later also shown in isolated human ventricular muscle strips were mediated by stimulation of the cardiac 5-HT_4_-serotonin receptor (Kaumann et al. 1990, Kaumann and Sanders 1994, Brattelid et al. 2004). Now, the access to human cardiac specimens is always limited. These muscle strips cannot be taken from cadavers but must be obtained from samples in the surgical theater. Thereafter, the strips have to be used as rapidly as possible for contraction experiments but also for biochemical studies. Thus, one cannot easily plan such experiments in human cardiac tissue. Moreover, patients are never disease-free or drug-free. This is a potential concern, because the function of 5-HT_4_-serotonin receptor is shown to be drug-treatment-, age- and disease-dependent in the human atrium (Kaumann and Levy 2006). To obviate some of these problems, we have generated mice with cardiac specific overexpression of human 5-HT_4_-serotonin receptor (5-HT_4_-TG). These mice are disease-free and are not drugged. Hence, it is easier to plan experiments with these transgenic mice than with human cardiac specimens. Admittedly, these transgenic mice also show drawbacks and limitations. While we overexpressed the human 5-HT_4_-serotonin receptor, we only expressed one splice variant. Many more splice variants are present in the healthy human heart and all of them probably mediate the effects of 5-HT (e.g., Brattelid et al. 2004). Moreover, our transgenic mouse model shows a much higher cardiac expression of the 5-HT_4_-serotonin receptor than noted in the human atrium (Gergs et al. 2009, Gergs et al. 2010). This is clear from the more potent positive inotropic effect of serotonin in left atrial preparations from 5-HT_4_-TG compared with human atrial preparations (Table 4).

Interestingly, the positive inotropic effects for some drugs of interest became in our studies only visible in isolated atrial preparations from 5-HT_4_-TG and/or man, when we impeded cAMP degradation. Others noted that the fading (decline in force over time) of the positive inotropic effects of gastrointestinal drugs was attenuated by pretreatment of atrial muscle strips by phosphodiesterase inhibitors (e.g., 3-isobutyl-1-methyl-xantine, IBMX, pig atria: De Maeyer et al. 2008). The purpose of phosphodiesterase inhibitors in all cases was to amplify the cAMP levels in the cardiac preparations and thus tilt the scales to exert a detectable positive inotropic effect for the drug of interest (Fig. 1A, Table 4). This was done with rolipram in mouse atrium but with cilostamide in human atrium (e.g., Neumann et al. 2024b). Hence, when we applied phosphodiesterase inhibitors, we probably overestimated the potency and effectivity of the drugs of interest in the 5-HT_4_-TG and possibly also in the human atrium. On the one hand, there is the benefit that the atrium of the wild type mouse (WT) does not respond to serotonin and hence does not react with the drugs of interest via serotonin receptors. On the other hand, if we detect a contractile effect in the atrium of WT, this indicates it is not 5-HT_4_-serotonin receptor-mediated but due to inhibition or stimulation of other receptors or ion channels or monoamine transporters or release of endogenous compounds, e.g., of noradrenaline from cardiac stores (cf. Table 1 for ancillary effects). On the one hand, there should always be an effect in 5-HT_4_-TG if drugs are agonists (or antagonists) at 5-HT_4_-serotonin receptors. On the other hand, if a drug has not have an effect in 5-HT_4_-TG it is not expected that any observed effect on force of contraction of this drug in the human atrium is mediated by 5-HT_4_-serotonin receptors. Some examples are summarized in Table 4. Based on the mouse data, we then performed studies in human atrium. Guided by the findings in mice, it was easier to design definite experiments in human atrium. In human atrium, we usually noted a similar qualitative pattern of agonism or antagonism of these drugs (Fig. 1A) as in atrium from 5-HT_4_-TG. Hence, for some drugs (Table 4) an action in the patient is less probable because the patients should have taken phosphodiesterase inhibitors to detect any cardiac effects with the drugs of interest. Now, there are drugs used in cardiology (e.g., levosimendan) that act as phosphodiesterase inhibitors. Moreover, there are drinks or food that contain phosphodiesterase inhibitors (e.g., caffeine). In addition, drug/drug interactions may increase plasma levels of drugs. Examples are given in Table 3. Such interactions may rise plasma levels of the drugs of interest and augment their inotropic effect in the atrium of patients.

**Table 4:** Synopsis of the stimulatory action of the drugs of interest on cardiac preparations

Drug	5-HT_4_ in vitro	RA 5-HT_4_ TG	Porcine RA	LA-5-HT_4_ TG	Porcine LA	HAP	HAP + PDE
Bromopride	ND	6.0^14^	ND	6.0^14^	ND	NE	< 6.0^14^
Cinitapride	ND	NCE^16^	ND	NIE^16^	ND	NE^16^	< 6.0^16^
Cisapride	6.8^7^ 7.4^1,8^	8.3^9^	6.4^10,11^	7.3^9^	NE^13^	< 6.1^21^ 6.3^13^ 6.9^7^	ND
Clebopride	ND	6.0^15^	ND	6.3^15^	ND	NE	< 6.0^15^
Felcisetrag	9.3^3^	10.2^25^	ND	9.7^25^	ND	NE	8.3^15^
Metoclopramide	6.7^1^	6.1^17^	5.7^11^	6.0^17^	ND	< 6.0^17^	ND
Mosapride	7.2^1^	< 6.0^18^	ND	> 6.0^18^	ND	NE^18^	< 6.0^18^
Naronapride	5.9^2^ 6.5^7^	7.3^19^	ND	7.0^19^	ND	NE^19^	< 6.0^19^
Prucalopride	8.1^7^ 7.5^1^	8.3^9^	7.4^20^ 7.0^12^	7.0^9^	7.2^20^ 7.7^12^	7.2^20^	ND
Renzapride	6.7^6,8^	ND	6.7^11^ 6.8^10^	ND	6.8^10^	6.3^21^	ND
Serotonin	7.0^8^	8.3^9^	6.5^12^ 7.1^11^ 7.2^10^	7.3^9^	7.7^12^	6.6^22^	ND
Tegaserod	8.0^7^ 8.1^1^	< 6.0^24^	6.6^12^	< 6.0^24^	ND	6.9^7^	< 6.0^24^
Tiapride	ND	ND	ND	ND	ND	ND	ND
Velusetrag	7.7–8.3^5^	< 6.0^19^	ND	< 6.0^19^	ND	6.0^19^	7.0^19^
Zacopride	5.95^4^	7.02^3^	5.3–6.4^11^	< 6.0^23^	ND	< 6.0^23^	< 6.0^23^

The first column indicates the drugs of interest. The second column indicates the binding to the 5-HT_4_-serotonin receptors or the formation of cAMP in cells transfected with the 5-HT_4_-serotonin receptors. The third column indicates the negative decadic logarithm of half maximally effective concentrations of the drugs of interest for their positive chronotropic effect in isolated right atrial preparations from 5-HT_4_-TG, for their positive chronotropic effect in isolated right atrial preparations from pigs via 5-HT_4_-serotonin receptors, for their positive inotropic effect in left atrial preparations from 5-HT_4_-TG, for their positive inotropic effect in left atrial preparations in pigs, for their positive inotropic effect when given alone in right human atrial preparations (HAP) or finally for their positive inotropic effect when given in the presence of phosphodiesterase inhibitors (PDE) in right human atrial preparations (HAP). It is known that serotonin exerts positive chronotropic effects in a concentration dependent manner in isolated spontaneously beating atrial preparations from pigs and 5-HT_4_-TG (Medhurst and Kaumann 1993, Gergs et al. 2013). In many contractile studies the EC_50_-values could not be calculated because no plateau was reached. However, e.g., < 6.0 indicates that the EC_50_-value must be higher than 1 µM under the experimental conditions given in the references. *NCE* negative inotropic effects, *ND* not done, *NE* no effect, *NIE* negative inotropic effects

References: 1: pK_i_: Vickery et al. 2007, 2: Bowersox et al. 2011, 3: Beattie et al. 2011, 4: pEC for cAMP production: Dumuis et al. 1989, 5: pK_i_ = 7.7–8.3: Smith et al. 2008, Long et al. 2012, 6: pKi: Bach et al. 2001, 7: Chai et al. 2012, 8: Bach et al. 2001, 9: Keller et al. 2018, 10: Kaumann 1990, 11: for (+) and (-) zacopride respectively: Medhurst and Kaumann 1993, 12: De Maeyer et al. 2006 ND: not done 13: Conlon et al. 2018: NE: no inotropic effect, 14:Abella et al. 2025a, 15: Abella et al. 2025b, 16: Neumann et al. 2024c. 17: Neumann et al. 2021, 18: Neumann et al. 2024b, 19: Schmidt et al. 2025, 20: Krobert et al. 2005, 21: Kaumann et al. 1991, 22 Kaumann et al. 1990, 23: Neumann et al. 2024a, 24: Hesse et al. 2024, 25: Abella et al. 2025c, *NCE* negative chronotropic effect: acting as an antagonist, *NIE* negative inotropic effect: acting as an antagonist

### Cardiac serotonin receptors in the ventricles

Now, the question arises how ventricular function is affected by 5-HT_4_-serotonin receptors, because the left ventricle mainly determines the cardiac output and not the left atrium or the right atrium of the heart. This has been investigated but this issue is still not perfectly understood. To summarize the current view, in the healthy human adult ventricle 5-HT_4_-serotonin receptors play no role for force generation (Brattelid et al. 2004, Afzal et al. 2008). However, in severe human heart failure, one finds a higher expression of 5-HT_4_-serotonin receptors in the human ventricle and a positive inotropic effect of serotonin (Brattelid et al. 2004). In the failing human ventricle in the presence of cilostamide or 3-isobutyl-1-methylxanthine (an unselective phosphodiesterase-inhibitor), serotonin exerted a convincing positive inotropic effect in vitro (Brattelid et al. 2004, Afzal et al. 2008. review: Neumann et al. 2023a, b). Hence, in the human failing ventricle, agonistic or antagonistic effects of drugs acting on 5-HT_4_-serotonin receptors are to be expected.

### Serotonin receptors and arrhythmias

A relevant area of interest in cardiology is the role of serotonin for the genesis of atrial and ventricular arrhythmias (Kaumann and Sanders 1994; Brattelid et al. 2004, Christ et al. 2014). One knows that all cAMP-increasing agents have the danger of inducing cardiac arrhythmias (Kaumann and Sanders 1994). Now, serotonin (and cisapride) increased cAMP in the isolated human atrium (Kaumann et al. 1991). Hence, cAMP may have caused the arrhythmias seen under serotonin treatment in some patients (Kaumann and Sanders 1994, Gergs et al. 2009). Regardless of the underlying mechanism, there is convincing experimental evidence that serotonin can cause atrial arrhythmias in humans. For instance, in isolated human atrial preparations, serotonin could induce arrhythmias in a concentration-dependent fashion which could be reversed by antagonists at 5-HT_4_-serotonin receptors (Kaumann and Sanders 1994). Further evidence comes from an animal model. We have overexpressed the human 5-HT_4_-serotonin receptors in cardiomyocytes in mouse hearts (5-HT_4_-TG). In isolated right atrial preparations from these 5-HT_4_-TG but not in wild-type littermates, serotonin induced in a concentration-dependent fashion a positive chronotropic effect (Gergs et al. 2010, Gergs et al. 2013, Keller et al. 2018). Moreover, like in the human atrial preparations (Kaumann and Sanders 1994), also in isolated right atrial preparations from 5-HT_4_-TG but not from wild-type littermates, serotonin induced in a concentration-dependent manner arrhythmias that were reversed by antagonists at 5-HT_4_-serotonin receptors (Gergs, et al. 2010, Gergs et al. 2013, Keller et al. 2018). Also, in a pig heart model, in vivo, one found that pacing-induced atrial arrhythmias could be reversed by an experimental antagonist at the 5-HT_4_-serotonin receptor (Rahme et al. 1999). Thus, in principle, drugs that are antagonists at the 5-HT_4_-serotonin receptor may turn out to be useful as antiarrhythmic agents (Rahme et al. 1999). Here might be a window of opportunity for some of these drugs (Fig. 1B): They are usually long used in the clinic and their dangers were understood over the years. Hence, the safest of them with antagonistic effect could be well be tried in the clinic to stop supraventricular arrhythmias. We might mention here also clinically studied 5-HT_4_-serotonin receptor antagonists such as piboserod and tropisetron. Now, 5-HT_4_-serotonin receptors are present and functional in the human atrium (and human ventricle (Kaumann and Levy 2006)). In animals, only pig and monkey hearts express functional 5-HT_4_-serotonin receptors (review: Neumann et al. 2021). This receptor stimulation leads to positive inotropic effects (Kaumann and Levy 2006). Interestingly, 5-HT_4_-serotonin receptor agonists may be useful to treat depression and Morbus Alzheimer (Lanthier et al. 2020). Indeed, we recently reported on such an investigational drug (Neumann et al. 2024a, b, c, d). A caveat is in order. Stimulation of 5HT_4_-serotonin receptors in the human heart can lead to arrhythmias and as such would be detrimental (Kaumann and Sanders 1994). Cardiac 5-HT_4_-serotonin receptors could be of clinical relevance: for one, 5-HT_4_-serotonin receptors exist on human cardiomyocytes. They (Fig. 1A) stimulate adenylyl cyclase and are thereby functional in the human heart (Dumuis et al. 1989). In the isolated muscle strips or cardiomyocytes from right and left human auricles, these 5-HT_4_-serotonin receptors augment the current through the L-type calcium cation channels (Kaumann and Levy 2006, Ouadid et al. 1992, Jahnel et al. 1993). In addition, they increase the phosphorylation state of cardiac regulatory proteins in human cardiomyocytes (Gergs et al. 2009, Christ et al. 2014). Only pig and monkey atria, but not atria from rat, rabbit, dog, or normal mouse, display a 5-HT_4_-serotonin receptor-mediated increase in beating rate (Medhurst and Kaumann 1993, review: Neumann et al. 2023a, b).

## Introduction to cardiac effects of prokinetic compounds

While there is evidence that stimulation of 5-HT_4_-serotonin receptors in the gut of patients with, e.g., irritable bowel syndrome can improve symptoms and may reduce intestinal pain, at least cisapride and tegaserod have severe cardiac side effects that are at least worrisome or even led to the removal of these drugs from many markets or led to a reduction of possible indications. These cardiac side effects were usually explained as off-target effects; implying that the proper targets are 5-HT_4_-serotonin receptors (Mawe and Hoffman 2013, Table 1). The assumption was and may still be that new prokinetic drugs need to be developed that should be selective for 5-HT_4_-serotonin receptor and these drugs would therefore be devoid of cardiac side effects mediated by other receptors (Mawe and Hoffman 2013). Another proposal to reduce cardiac side effects was to devise 5-HT_4_-serotonin receptor agonists that stay in the bowel; in other words have a peroral bioavailability of zero (Mawe and Hoffman 2013). We will address the gastrointestinal drugs in alphabetical order. We have labeled in Table 1 with * those drugs which are approved drugs in humans, at least in some jurisdictions.

### Bromopride

Bromopride (2-methoxy-4-amino-5-bromo-N,N-diethylaminoethylbenzamide, Fig. 1B) is a structural analogue to metoclopramide (2-methoxy-4-amino-5-chloro-N,N-diethylaminoethylbenzamide, Fig. 1B, Epifanio et al. 2018, Suffredini et al. 2010). Bromopride was initially developed together with metoclopramide in search for analogues of procainamide (Fig. 1B). Bromopride, in other words, was initially intended to be used as antiarrhythmic agent (Mouillé et al. 1977). However, bromopride had no impressive antiarrhythmic effects in animal studies (living dogs were used). Per oral bromopride is currently used as a prokinetic drug in the gastrointestinal tract of patients (Tonini et al. 2004). Bromopride has antagonistic effects at central and intestinal D_2_-dopamine receptors (Meltzer et al. 1983, Epifanio et al. 2018, Table 3). Indeed, the known antiemetic action of bromopride is explained by block of D_2_-dopamine receptor in the area postrema of the human brain (Tonini et al. 2004). For instance, bromopride reduced nausea in cancer patients receiving emetogenic anti-cancer drugs (Roila et al. 1985; Dunne et al. 2013; Table 3). Bromopride is metabolized in humans, mainly in the liver, but it is uncertain which isoform of the cytochromes is involved (Dunne et al. 2013). Several oxidative and glucuronide metabolites of bromopride have been reported in humans (Dunne et al. 2013). Bromopride increased force and beating rate as an agonist at cardiac human 5-HT_4_-serotonin receptors because bromopride only increases contractility in atrium from 5-HT_4_-TG and not in WT (Table 4). One can conclude that bromopride acts at 5-HT_4_-serotonin receptors in left and right atrium of 5-HT_4_-TG as a partial agonist. Bromopride furthermore stimulated 5-HT_4_-serotonin receptors in the isolated human atrium (Table 4, Table 4). We would predict that a tachycardia after treatment with bromopride in patients could occur.

### Cinitapride

Cinitapride (4-amino-N-[1-(3-cyclohexen-1-ylmethyl)−4-piperidinyl]−2-ethoxy-5-nitrobenzamide, Fig. 1B) is used to treat dyspepsia and gastroesophageal reflux disease (Marquez et al. 2011). It is metabolized by cytochromes (Campodonico et al. 2022). Cinitapride is also used as an antiemetic agent. Moreover, after serotonin was given to increase force of contraction, cinitapride reduced force of contraction in human right atrial preparations. This suggests that cinitapride is also an antagonist at human 5-HT_4_-serotonin receptor. Similarly, cinitapride alone reduced the beating rate in spontaneously beating isolated right atrial preparations from 5-HT_4_-TG. If one assumes that 5-HT_4_-TG recapitulates the situation in the human sinus node, then this indicates that cinitapride could have a negative chronotropic in patients. Cinitapride alone minimally augmented force of contraction in human right atrial preparations. However, in the presence of cilostamide, a phosphodiesterase 3 inhibitor, cinitapride has a positive inotropic effect in human right atrial preparations. This effect is reversed by GR125487, a selective antagonist at 5-HT_4_-serotonin receptors (Schiavi et al. 1994; Neumann et al. 2024c). GR125487 is an indole derivate (Fig. 1B). GR125487 has a very high affinity (0.19 nM) for 5-HT_4_-serotonin receptors, for instance in the brain (Schiavi et al. 1994). This is an argument that cinitapride is an agonist at 5-HT_4_-serotonin receptors. Cinitapride has been tried with some success to treat gastroparesis (Camilleri and Atieh 2021).

### Cisapride

Cisapride ((3*RS*,4*SR*)−4-Amino-5-chlor-*N*-{1-[3-(4-fluorphenoxy)propyl]−3-methoxypiperidin-4-yl}−2-methoxybenzamide, Fig. 1B) was not developed as a 5-HT_4_-serotonin receptor agonist. More by chance, cisapride was noted to stimulate (with a similar potency as serotonin but with a higher efficacy) cAMP formation by augmenting the activity of adenylyl cyclase in the brain of mouse embryos or of adult guinea pigs (Dumuis et al. 1989). As mentioned above, the receptor that led to this increase in cAMP was called 5-HT_4_-serotonin receptor in order to distinguish it from the hitherto known 5-HT_1_-, 5-HT_2_- and 5-HT_3_-serotonin receptors (Dumuis et al. 1988, Dumuis et al. 1989). Cisapride has the advantage compared to the older drug metoclopramide that it does not block D_2_-dopamine receptors and therefore does not exert extrapyramidal side effects. However, due to reports of a constriction of coronary arteries and increases in arrhythmias, cisapride usage was greatly inhibited by regulators (Potet et al. 2001). Cisapride alone exerted hardly a positive inotropic effect in left atrial preparation of human 5-HT_4_-TG (Table 4). Interestingly, this positive inotropic of cisapride was more efficient when cisapride was applied in left atrial preparations from mice with cardiac specific overexpression of the human mutated D100A-TG (congress abstract: Gergs and Neumann 2025). In these mice, we expressed a mutated 5-HT_4_-serotonin receptor, i.e., D100A. A single amino acid was exchanged: aspartic acid at position 100 of the receptor was mutated to the amino acid alanine (Gergs and Neumann 2025). In these mice, D100A-TG, serotonin had no positive inotropic effect in isolated left atrial preparations (Gergs and Neumann 2025). This finding is in line with previous biochemical studies. One has transfected HEK293 cells with the normal receptor (for brevity D100D) or the mutated receptor (D100A). In these cells, serotonin increased cAMP levels only in D100D-5-HT_4_-serotonin receptor (i.e., wild-type receptor)-transfected cells but not in D100A-5-HT_4_-serotonin receptor (mutated receptor)-transfected cells (Chang et al. 2007). In contrast, cisapride raised cAMP-levels in D100A-receptor transfected cells. Assuming that cardiomyocytes behave similarly, one could explain that cisapride raised force of contraction in atrial preparations from D100A-TG because in these atria it hypothetically might have raised cAMP levels (Gergs and Neumann 2025). This contraction study is preliminary functional evidence that cisapride does not require the aspartic acid at position 100 in the sequence of the 5-HT_4_-serotonin receptor to stimulate the receptor to generate cAMP and thus increase force of contraction. Cisapride has been tried with some success to treat gastroparesis (Camilleri and Atieh 2021). Due to arrhythmias (palpitations, tachyarrhythmias, torsade de pointes, ventricular fibrillation and sudden cardiac death, e.g., Smalley et al. 2000), usually explained by an inhibition of voltage-gated inwardly rectifying potassium channels (human Ether-à-go-go-Related Gene, hERG, Table 1), cisapride is currently available only in some countries and only for selected patients (Camilleri and Atieh 2021). Cisapride has been studied in many gastrointestinal mobility disorders but the evidence of a benefit was probably best in the treatment of nocturnal heartburn (Tack et al. 2012). Cisapride is metabolized by CYP3A (Table 3) and thus inhibitors (like erythromycin) of this enzyme will increase plasma concentrations and thus cardiac side effects of cisapride (Tack et al. 2012). Serotonin seems to bind to another part of the 5-HT_4_-serotonin receptors than benzamides like cisapride: cisapride binds to the D100A-(= D3.32A) mutated 5-HT_4_-serotonin receptor, whereas serotonin does not bind to this mutated receptor (Claeysen et al. 2003, Bockaert et al. 2008, Pellissier et al. 2009). It has been suggested that cisapride binds to threonine 104 (T.3.36) on the 5-HT_4_-serotonin receptor (Bockaert et al. 2008; Pellissier et al. 2009). This seems plausible because in cells transfected with the properly mutated 5-HT_4_-serotonin receptors, namely, T104A (T3.36A), serotonin could raise cAMP concentrations while cisapride did not augment cAMP concentrations (Pelissier et al. 2009). We are not aware whether mutations such as D100A or T104A of the 5-HT_4_-serotonin receptor exist in humans. Cisapride was a partial agonist at low expressional levels in transfected cells for the human 5-HT_4b_-serotonin receptors which probably mimics the situation in the human atrium (Bach et al. 2001). In contrast, cisapride was a full agonist at high expressional levels of the 5-HT_4b_-serotonin receptor (Bach et al. 2001). One can conclude that cisapride acts at 5-HT_4_-serotonin receptors in left and right atrium of 5-HT_4_-TG as a partial agonist. Cisapride furthermore stimulated 5-HT_4_-serotonin receptors in the isolated human atrium (Table 1, Table 4).

### Clebopride

Clebopride (4-amino-N-(1-benzylpiperidin-4-yl)−5-chloro-2-methoxybenzamide) was developed to block gut D_2_-dopamine receptors more potently than metoclopramide (Tonini et al. 2004). In some countries, clebopride is an approved anti-dopaminergic gastrointestinal prokinetic drug. Its effects on the gut are thought to result from both an inhibition D_2_-dopamine receptors and a stimulation of 5-HT_4_-serotonin receptors in the gut (Tack et al. 2012). In addition, clebopride has anti-emetic effect via antagonism of brain D_2_-dopamine receptors and brain 5-HT_3_-serotonin receptors (Tack et al. 2012). Clebopride might be useful to treat gastroparesis and postoperative nausea (Duarte et al. 1985, Camilleri and Atieh 2021). While clebopride inhibited the hERG this occurred at plasma concentrations that are much higher than under normal conditions (Tack et al. 2012). Clebopride is metabolized via CYP3A4 (Table 3) and thus interactions are conceivable (Tack et al. 2012). Clebopride increased force of contraction and beating rate in isolated atria of 5-HT_4_-TG (Table 4). In addition clebopride, also increased force of contraction in the isolated human atrium (Table 4, Rayo Abella et al. 2025a, b, c).

### Felcisetrag

Felcisetrag is a potent ligand at 5-HT_4_-serotonin receptors (Beattie et al. 2011, Table 4). Felcisetrag (Fig. 1B: methyl 4-[[4-[[(2-propan-2-yl-1*H*-benzimidazole-4-carbonyl)-amino]methyl]piperidin-1-yl]methyl]piperidine-1-carboxylate) can be structurally regarded as a derivative of serotonin (McKinnell et al. 2013, Fig. 1B). Felcisetrag is not yet approved for patients. Felcisetrag has gastrointestinal prokinetic properties in animals and in humans (Beattie et al. 2011). Felcisetrag is rather specific (Beattie et al. 2011, Table 1). This may be an advantage of felcisetrag compared to its predecessors (Beattie et al. 2011). There are at present eight completed or still running clinical trials in www.clinical trials.gov for felcisetrag. One study addressed the intestinal mobility in patients after gut surgery (Boeckxstaens et al. 2024). Felcisetrag was active against gastroparesis (Chedid et al. 2021). Felcisetrag is mainly eliminated by the kidney. Felcisetrag is metabolized in a minor part via the cytochrome CYP3A4 (Chen et al. 2022, Table 3). Hence, one can understand why inhibitors of CYP3A4 like itraconazole were found to inhibit the metabolism of felcisetrag and why itraconazole was noticed to elevate the plasma concentration of felcisetrag (Chen et al. 2022, Czerniak et al. 2022; Pusalkar et al. 2022). Because felcisetrag is mainly eliminated via the kidney, one would assume that in patients with kidney disease, the plasma levels of felcisetrag might be elevated and might cause more side effect in the heart. Felcisetrag has the advantage over other prokinetic drugs like cisapride that it did not lead to vasoconstriction of isolated human coronary arteries (Beattie et al. 2011). Felcisetrag raised force of contraction and beating rate in isolated atria of 5-HT_4_-TG (Table 4). In addition felcisetrag, also increased force of contraction in the isolated human atrium (Table 4).

### Metoclopramide

Metoclopramide (2-methoxy-4-amino-5-chloro-N,N-diethylaminoethylbenzamide, Fig. 1B) was probably the first 5-HT_4_-serotonin receptor agonist that was useful to treat reduced mobility of the gut (De Maeyer et al. 2008, Tack et al. 2012). Metoclopramide was less potent and less effective than serotonin to stimulate via 5-HT_4_-serotonin receptors the formation of cAMP in mouse brain preparations (Dumuis et al. 1989). This formation of cAMP was competitively antagonized by tropisetron (at that time called ICS 205 930: Dumuis et al. 1989). We know now that tropisetron is both a 5-HT_3_-serotonin receptor antagonist and a 5-HT_4_-serotonin receptor antagonist (Dumuis et al. 1989, Barnes et al. 2021). Metoclopramide is approved in the USA for the treatment of gastroparesis and is also used as anti-emetic drug. The blockade of central D_2_-dopamine and/or 5-HT_3_ serotonin receptors is thought to underlie the anti-emetic effects of metoclopramide (Wallenborn et al. 2006). The central D_2_-dopamine receptor blockade by metoclopramide can lead to increased prolactin levels in the plasma and also to Parkinson-like side effects (Ohno et al. 2003). This has led in many countries to restrictions in the use of metoclopramide. Metoclopramide is a partial agonist at 5-HT_4_-serotonin receptors in the human atrium (Neumann et al. 2021).

### Mosapride

Mosapride (4-amino-5-chloro-2-ethoxy-N-[[4-[(4-fluorophenyl)methyl]−2-morpholinyl]methyl]benzamide, Fig. 1B) has been tried with some success to treat especially gastroparesis (Camilleri and Atieh 2021). In ligand binding studies mosapride was an agonist at 5-HT_4_-serotonin receptors and an antagonist at 5-HT_3_-serotonin receptors (Katoh et al. 2003, Tsubouchi et al. 2018). Mosapride was developed with the aim to design a drug that like metoclopramide stimulates 5-HT_4_-serotonin receptors but does not block D_2_-dopamine receptors in the brain that may cause extrapyramidal side effects (Katoh et al. 2003). Mosapride has an active metabolite (des-fluorobenzyl-mosapride, Katoh et al. 2003). Mosapride is degraded by CYP3A4 opening the possibility of drug interactions with, e.g., erythromycin. Mosapride has the advantage not to inhibit hERG at therapeutic drug concentrations (Katoh et al. 2003, Tack et al. 2012). Furthermore, it turned out that mosapride increased force of contraction in left atria from 5-HT_4_-TG and augmented the heartbeat in right atrial preparations from 5-HT_4_-TG (Table 4). Moreover, mosapride but also its main metabolite, des-fluorobenzyl-mosapride, elevated force of contraction in isolated human atria (Table 4).

### Naronapride

Naronapride (initially called ATI-7505, [(3R)−1-azabicyclo[2.2.2]octan-3-yl] 6-[(3S,4R)−4-[(4-amino-5-chloro-2-methoxybenzoyl)amino]−3-methoxypiperidin-1-yl]hexanoate, Fig. 1B) is an experimental drug. Naronapride was devised as a selective, high affinity (Ki = 1.4 nM) agonist at 5-HT_4_-serotonin receptors in ligand binding studies (Bowersax et al. 2011). Naronapride has been tested with success to treat gastrointestinal diseases, such as gastroparesis in patients. Naronapride was further investigated because it has the advantage that it hardly inhibits the hERG channel (Bowersax et al. 2011, Table 1). Naronapride is metabolized by cytochromes, but only to a small extent (Bowersax et al. 2011, Table 1). Therefore, naronapride is unlikely to show interaction with other drugs metabolized by cytochromes (Bowersax et al. 2011). In HEK293 cells transfected with the human 5-HT_4*b*_-serotonin receptor, naronapride increased the cAMP content of these cells. In other words, naronapride behaved biochemically as an agonist at the human 5-HT_4*b*_-serotonin receptor. Naronapride was designed to be metabolized by ubiquitous carboxylesterases (Bowersax et al. 2011). In a clinical phase I study, naronapride exhibited a half-life of about 6 h (Bowersox et al. 2011, Table 1). A peak plasma concentration of 24 nM was reached 2 h after applying 120 mg naronapride per os (Bowersox et al. 2011, Table 1). Naronapride increased force of contraction in left atria from 5-HT_4_-TG and augmented the heartbeat in right atrial preparations from 5-HT_4_-TG (Table 4). Moreover, naronapride raised force of contraction in human atria (Neumann et al. 2024c; Schmidt et al. 2025; Table 4).

### Prucalopride

Prucalopride (4-amino-5-chlor-*N*-[1-(3-methoxypropyl)piperidin-4-yl]−2,3-dihydrobenzofuran-7-carboxamide, Fig. 1A, B) is rather specific for 5-HT_4_-serotonin receptors. For instance, prucalopride seems not to inhibit hERG currents (Tack et al. 2012). In this way, prucalopride should not prolong the duration of action potentials and should not lead as a consequence to arrhythmias via this mechanism. Of note, prucalopride did not block 5-HT_1_-, 5-HT_2A_-, 5-HT_2B_-, 5-HT_3_-serotonin receptors and did not exhibit anti-cholinergic effects and did not inhibit the activity of cholinesterase (Briejer et al. 2001). Prucalopride is chemically a dihydrobenzofurancarboxamide and thus not a benzamide in contrast to most other compounds discussed here (Frampton 2009, Fig. 1B). Prucalopride showed some selectivity for splice variants of the 5-HT_4_-serotonin receptors in cell culture (human embryonic kidney 293 cells): prucalopride was more potent, for instance, to raise Ca^2+^ transients in cells that expressed 5-HT_4B_-serotonin receptors than in cells that expressed 5-HT_4A_-serotonin receptors (Pindon et al. 2002). Prucalopride increased force of contraction in left atria from 5-HT_4_-TG and augmented the heartbeat in right atrial preparations from 5-HT_4_-TG (Table 4). Moreover, prucalopride increased force of contraction in human atria (Table 4).

### Renzapride

Renzapride (4-amino-N-[(4R,5S)−1-azabicyclo[3.3.1]nonan-4-yl]−5-chloro-2-methoxybenzamide) is an agonist at isolated 5-HT_4_-serotonin receptors and an antagonist at isolated 5-HT_3_-serotonin receptors. Renzapride is an agonist at animal gut preparations via 5-HT_4_-serotonin receptors (Buchheit and Buhl 1991). Renzapride is a racemic drug. As an exception to the general rule, the enantiomers R- and S-renzapride exhibit a similar pharmacology (Meyers and Hickling 2008). The affinity of renzapride at the 5-HT_3_ serotonin receptor (17 nM) was much higher than at the 5-HT_4_-serotonin receptor (around 140 −450 nM) in ligand binding studies at recombinant guinea pig receptors (Meyers and Hickling 2008). Other receptors were not bound by renzapride or with irrelevant affinity, namely, e.g., adrenergic receptors, dopamine receptors or 5-HT_1_-serotonin receptor or 5-HT_2_-serotonin receptors (Table 1). Renzapride had nearly no affinity for dopamine, serotonin or catecholamine transporters (Meyers and Hickling 2008). Renzapride has renzapride N-oxide as an active metabolite which is formed by CYP3A4. Renzapride itself could inhibited the activity of several isoenzymes namely CYP1A2, CYP2A6 6.9%, CYP2C9, CYP2C19, CYP2E1, and CYP3A4 (Table 1, Meyers and Hickling 2008). Renzapride was a full agonist at the recombinant 5-HT_4b_-serotonin receptor and a partial agonist at recombinant 5-HT_4a_-serotonin receptors (Blondel et al. 1998). Renzapride was never approved as a drug probably because its clinical benefit was too small (Tack et al. 2012). Like cisapride, also renzapride was a partial agonist at low expressional levels for the human 5-HT_4b_-serotonin receptors in transfected cells: this probably mimics the situation in the human atrium (Bach et al. 2001). However, renzapride was a full agonist at high expressional levels of the 5-HT_4b_-serotonin receptor (Bach et al. 2001). Moreover, renzapride increased force of contraction in human atria (Table 4).

### Tegaserod

Tegaserod (2-[(5-methoxy-1H-indol-3-yl)methylene]-N-pentyl-hydrazinecarboximidamide, Fig. 1B) inhibits also the transporters for serotonin (SERT) or dopamine (DAT) or noradrenaline (NET) (Ismair et al. 2007). For fear of vasoconstriction in coronary arteries in patients, the sale of tegaserod had been suspended for some time (Thompson 2007). Tegaserod seems to bind to another part of the 5-HT_4_-serotonin receptor than benzamides like metoclopramide, renzapride and cisapride: Renzapride and cisapride bound to the D100A-(= D3.32A) mutated 5-HT_4_-serotonin receptor and tegaserod does not (Clayesen et al. 2003, Bockaert et al. 2008, Pellissier et al. 2009). Hence, serotonin and tegaserod seem to bind to different amino acids than benzamides. As mentioned above, benzamides bind to T.3.36 on 5-HT_4_-serotonin receptors (Bockaert et al. 2008; Pellissier et al. 2009). Hence, one would predict that tegaserod like serotonin would not exert a positive inotropic effect in left atrial preparations from D100A-TG, but this has not yet been studied. Tegaserod exhibits a high affinity at 5-HT_4_-serotonin receptors. Tegaserod also exhibited some affinity for 5-HT_2_- and 5-HT_1_-serotonin receptors (Tack et al. 2012). The vasoconstriction by tegaserod in human coronary arteries has been explained by a stimulation of these 5-HT_1_-serotonin receptors. Due to these cardiac side effects, tegaserod was removed from the market in the USA but under restrictions re-entered the market later. Tegaserod was never approved in Europe (Tack et al. 2012). Tegaserod has a proven benefit in certain forms of irritable bowel disease (Tack et al. 2012). Tegaserod increased force of contraction in left atria from 5-HT_4_-TG and augmented the heartbeat in right atrial preparations from 5-HT_4_-TG (Table 4). Moreover, tegaserod increased force of contraction in human atria (Table 4).

### Velusetrag

Velusetrag (N-[(1R,5S)−8-[(2R)−2-hydroxy-3-(methyl-methylsulfonylamino)propyl]−8-azabicyclo[3.2.1]octan-3-yl]−2-oxo-1-propan-2-ylquinoline-3-carboxamide, Fig. 1B) is an indazole–tropane and not a benzamide (Long et al. 2012). A whole series of indazole–tropanes was synthesized and optimized for good oral resorption; this process led to velusetrag. Velusetrag binds to (pK_i_ = 7.7–8.3) and stimulates 5-HT_4_-serotonin receptors in transfected Chinese hamster ovary cells to produce cAMP (Smith et al. 2008, Long et al. 2012). Hence, velusetrag is about as potent as its comparator tegaserod and more potent than older similarly acting drugs like cisapride or mosapride in such assays (Smith et al. 2008, Long et al. 2012). Moreover, velusetrag was similarly effective as serotonin to increase cAMP-levels in cells transfected with splice variants of the 5-HT_4_-serotonin-receptor but also in functional assays of guinea-pig colon preparations or rat esophageal preparations (Smith et al. 2008). Velusetrag increased in vivo the gastrointestinal motility in several animal species, like guinea pig, rat, mouse and dog (Beattie et al. 2008, Grigoletto et al. 2023). Velusetrag was more than 500-fold more selective for the 5-HT_4_-serotonin receptor compared to other serotonin receptors like the 5-HT_3_-serotonin receptor in vitro in ligand binding assays (Smith et al. 2008). Velusetrag is functionally nearly a full agonist (81% of serotonin) at guinea-pig intestinal 5-HT_4_-serotonin-receptors with regard to contraction (Smith et al. 2008). Velusetrag was inactive against many other GTP-binding-protein coupled receptors. For instance, velusetrag does not block, at least in relevant concentrations, 5-HT_3_-serotonin or D_2_-dopamine receptors (Smith et al. 2008): this is clinically important because velusetrag should not have antiemetic properties via 5-HT_3_-serotonin receptors and because velusetrag should not elicit Parkinson-like symptoms via D_2_-dopamine receptors. The only relevant interaction in ligand binding studies showed velusetrag at H_1_-histamine receptors (pK_i_ = 6.3). Moreover, in the guinea-pig trachea, a model system for the function of H_1_-histamine receptors, 30 µM velusetrag produced a small rightward shift of the concentration response curve of histamine (1.8-fold shift of the EC_50_-value, Smith et al. 2008) suggesting competitive antagonism the H_1_-histamine receptors. Whether this holds true in the human heart, remains to be studied. Remarkably, velusetrag inhibited only marginally (by 13%, at 3 µM) the hERG channels in vitro (Smith et al. 2008, Long et al. 2012, Beattie et al. 2013). In patients, velusetrag increased gastrointestinal mobility and was studied to treat chronic constipation (review: Zhang et al. 2020). Moreover, velusetrag improved gastric emptying in patients with diabetes (Abell et al. 2023). In that study no changes with highest dose of velusetrag tested (15 mg) were noted on heart rate, systolic or diastolic blood pressure. However, in some patients with gastroparesis, the QT interval in the ECG was somewhat prolonged (Abell et al. 2023). Velusetrag at 50 mg daily did not cause palpitations or other arrhythmias in humans in a phase II trial (Goldberg et al. 2010). However, there was an increase in the heartbeat (by 10 bpm) in patients with chronic constipation (Goldberg et al. 2008). Currently, one finds five clinical trials for velusetrag in clinical trials.gov. One indication currently tested for velusetrag is chronic intestinal pseudo-obstruction (clinical trials.gov). Velusetrag increased force of contraction in left atria from 5-HT_4_-TG and augmented the heartbeat in right atrial preparations from 5-HT_4_-TG (Table 4). Moreover, velusetrag increased contractility, intracellular Ca^2+^ transients and the current through the L-type calcium ion channel in ventricular cardiomyocytes from 5-HT_4_-TG (Pauls et al. 2026). Moreover velusetrag increased force of contraction in human atria (Table 4).

### Zacopride

Zacopride (4-amino-N-(1-azabicyclo[2.2.2]octan-3-yl)−5-chloro-2-methoxybenzamide) falls into the class of substituted benzamides (Fig. 1B). Its chemical structure is similar to that of renzapride. Zacopride was developed before 5-HT_4_-serotonin receptors were functionally recognized or cloned (Barnes et al. 1988). In fact, zacopride was developed as an antagonist at 5-HT_3_-serotonin receptors and intended to be used as an antiemetic drug (Barnes et al. 1988). In guinea pig colon and ileal strips, zacopride attenuated contractions induced by 5-HT (Eglen et al. 1993). This was the first evidence that zacopride acts as a functional partial antagonist at 5-HT_4_-serotonin receptors (Eglen et al. 1990, Elswood et al. 1991). Furthermore, in mouse embryo colliculi neurons, zacopride stimulated adenylate cyclase activity (pEC_50_-value = 5.95, Dumuis et al. 1989, Table 4). Zacopride was thus more potent than other benzamides, such as metoclopramide (pEC_50_-value = 5.34, Table 4), but less potent than the cisapride (pEC_50_-value = 7.14, Table 4) in this assay (Dumuis et al. 1989). Somewhat surprisingly zacopride was more effective than serotonin to raise cAMP in mouse brain preparations (Dumuis et al. 1989). Few clinical studies on zacopride ensued: an oral dose of 400 μg of zacopride was given by endocrinologists; at this dosage, zacopride did not alter blood pressure or heart rate in healthy volunteers (Lefebvre et al. 1993). Zacopride stimulated the current through L-type calcium ion channel in isolated atrial human cardiomyocytes, acting also here as an agonist at 5-HT_4_-serotonin receptors (Ouadid et al. 1992). Zacopride may directly act on 5-HT_4_-serotonin receptors in the sinus node: zacopride induced dose-dependent tachycardia in anesthetised pigs (Villavon et al. 1991, Eglen et al. 1993). Moreover, in this pig model, zacopride is a partial agonist: zacopride antagonized the tachycardia induced by 5-HT in pigs (Villalón et al. 1991). Interestingly, one noted acute negative inotropic effects in isolated human ventricular strips at 100 μM of zacopride (Elnakish et al. 2017). This is almost certainly a toxic concentration not reached in humans. However, we did not find pharmacokinetic data of zacopride in humans in the public domain. Nevertheless there is ongoing interest in zacopride in experimental cardiology, as a cardioprotective agent: probably, because zacopride elevates cAMP levels in cardiomyocytes, zacopride also activates by this pathway K1 potassium ion currents in the rat heart (Wu and Cao 2014, Zhai et al. 2017). It is unclear whether this pathway is also stimulated in the human atrium by zacopride. Zacopride is a full agonist in atria of 5-HT_4_-TG and a partial agonist in the isolated human right atrium via human 5-HT_4_-serotonin receptors. As already mentioned, serotonin seems to bind to another part of the 5-HT_4_ -serotonin receptor than benzamides like zacopride: zacopride binds to the D100A-(= D3.32A) mutated 5-HT_4_-serotonin receptor, whereas serotonin does not bind (Claeysen et al. 2003, Bockaert et al. 2008, Pellissier et al. 2009). Hence, serotonin and zacopride bind to different amino acids. Indeed, it has been suggested that S-zacopride binds to threonine 104 (T.3.36) on the 5-HT_4_-serotonin receptor (Bockaert et al. 2008; Pellissier et al. 2009). This seems plausible because in cells transfected with the properly mutated 5-HT_4_-serotonin receptors namely T3.36A, serotonin could raise cAMP concentrations while S-zacopride failed to do so (Pelissier et al. 2009). In agreement with these biochemical studies, in the left atrium of D100A-TG, where serotonin failed to alter force, zacopride exerted a positive inotropic effect (Gergs and Neumann 2025).

### Pharmacokinetic considerations

When one wants to present a synopsis of the drugs of interest, one could distinguish several groups on the basis of their pharmacodynamics and pharmacokinetics. The drugs of interest (Fig. 1B) are usually oxidized by the cytochromes. Some of them, as many other drugs, mainly or even exclusively by CYP3A4 or CYP2D6 (Table 3). Some of the drugs are substrates for other cytochromes (bromopride, cinitapride, cisapride, clebopride, felcisetrag, metoclopramide, mosapride, renzapride, tegaserod, tiapride, velusetrag). Others are at least poorly degraded by cytochromes (naronapride and prucalopride). Velusetrag is not degraded at all by cytochromes. From a practical point of view that means on the one hand in some cases co-administration of erythromycin or its derivatives (often used in intensive care units to stimulate motilin receptors and therefore preserve the gut motility of severely sick patients) cannot be co-administrated. The same holds true for the older antifungal agents (ketoconazole) often used in intensive care to treat fungal infection in severely ill patients. On the other hand in drugs that are not or poorly metabolized by cytochromes such drug/drug interaction are probably not a concern for patient care. One may also judge from Table 3 that the half-life widely varies. The clinical situation usually requires a long half-life of these drugs. When we address the pharmacodynamics, from a theoretical view as a pharmacologist one would prefer drugs that only act at one receptor and probably one would prefer a full agonist in our case at the 5-HT_4_-serotonin receptor. However, in the clinic, the needs may be different. If one wants to treat nausea and emesis, one might find it beneficial if the drug of choice also inhibits 5-HT_3_-serotonin receptors. It is more problematic, if the drug in consideration also blocks D_2_-dopamine receptors. This can lead to extrapyramidal side effects, especially in younger patients where conceivably the blood brain barrier is still immature or in patients co-medicated with inactive transporters in glial cells. The same concern arises when the dosage to the D_2_-dopamine blocking drug is chosen to high. Actions on 5-HT_1_-serotonin receptors can lead to vasocontraction of coronary arteries and thus to angina pectoris. This seems to be one mechanism why cardiac side effects sometimes occurred with tegaserod and cisapride. Hence, we would conclude that blockade selective agonism at 5-HT_4_-serotonin receptors seems to be preferable.

### Putative action on cardiac dopamine receptors

Moreover, one may ask whether drugs that inhibit D_2_-dopamine receptors may have side effects in the human heart. D_2_-dopamine receptors have been documented in all regions of the human heart by autoradiography (reviewed in Neumann et al. 2023b). We have recently generated mice with cardiospecific expression of the human D_2_-dopamine receptor (D_2_-TG: Neumann et al. 2026b). In the left atria of D_2_-TG, stimulation of D_2_-dopamine receptors reduced force of contraction (Neumann et al. 2026b). If we hypothesize that the same holds true in the human atrium, then stimulation of D_2_-dopamine receptors should lead to a negative inotropic effect in human atria. Hence, drugs such as metoclopramide might block such negative inotropic effects via D_2_-dopamine receptors but this needs to be tested experimentally. Moreover, there are D_2_-dopamine receptors in neuronal cells of the heart (Neumann et al. 2023b) and their function might also be altered with drugs like metoclopramide. Furthermore, drugs like donecopride, that inhibit the activity of cholinesterases, would be expected to raise acetylcholine levels in the heart. This is expected to lead to a negative inotropic effect via M_2_-muscarinic receptors in the human isolated atrium (e.g., Dhein et al. 2001). But this is also hypothetical. Together with metoclopramide also sulpiride, tiapride and sultopride were synthesized (Fig. 1B) and tested in animal studies in search for anti-arrhythmic agents (Fontaine and Reuse 1978). However, sulpiride, tiapride and sultopride have found indications in psychiatry in part as atypical antipsychotics due to their inhibitory action on D_2_-dopamine receptors or are used treat “tic” disorders (Morel 1983, Arima et al. 1986, Steele et al. 1993, Mauri et al. 1996, Fekete et al. 2021). In our hands, sulpiride, tiapride and sultopride were hardly agonists but potent antagonists for the contractile effects of serotonin in isolated atria from 5-HT_4_-TG and human atrial preparations (Neumann et al. 2024c). Interestingly, sulpiride has antiemetic properties in patients which are explained by an antagonism at brain D_2_-dopamine receptors (Cohen et al. 1999).

## Conclusions

One can deduce from Table 4 that cardiac effects of 5-HT_4_-serotonin agonists are probably a concern in those drugs that increase force of contraction in the absence of phosphodiesterase inhibitors (cisapride, metoclopramide, prucalopride, renzapride, tegaserod, velusetrag and zacopride). In contrast, cardiac effects of 5-HT_4_-serotonin agonists are probably not relevant in those drugs that increase force of contraction only in the presence of phosphodiesterase inhibitors (bromopride, cinitapride, clebopride, felcisetrag and mosapride). However, even there some caution might be prudent. Usually patients nowadays will be treated with phosphodiesterase inhibitors like levosimendan only in specialized heart failure clinics. However, in such patients caution with gastrointestinal drugs of Table 4 might still be prudent. Moreover, it is conceivable that also in the presence of enhanced plasma catecholamines (noradrenaline and adrenaline), the cardiac effects of 5-HT_4_-serotonin receptors agonists might become visible. Finally, all partial agonists are also antagonists at 5-HT_4_-serotonin receptors at least at higher drug concentrations. Under special conditions this mechanism may gain clinical relevance. For instance, if in hypertension, heart failure or atrial fibrillation, high cardiac concentration of serotonin exist, then 5-HT_4_-serotonin antagonists might attenuate cardiac effects of serotonin. In rare cases this might be beneficial because serotonin itself is arrhythmogenic and therefore 5-HT_4_-serotonin antagonists might terminate such arrhythmias.

### Summary

In summary, some drugs that are used to stimulate intestinal mobility and are agonists at 5-HT_4_-serotonin receptors are probably devoid of detrimental effects on cardiac contractility in humans. A few drugs however, merit special caution. However, in severe heart failure and in the presence of, e.g., levosimendan all drugs in Table 4 are better avoided.

## Data Availability

Does not apply.
